# Breakpoint delineation in 5p‐ patients leads to new insights about microcephaly and the typical high‐pitched cry

**DOI:** 10.1002/mgg3.957

**Published:** 2019-09-30

**Authors:** Samar N. Chehimi, Évelin A. Zanardo, José R. M. Ceroni, Amom M. Nascimento, Fabrícia A. R. Madia, Alexandre T. Dias, Gil M. N. Filho, Marília M. Montenegro, Jullian Damasceno, Thaís V. M. M. Costa, Yanca Gasparini, Chong A. Kim, Leslie D. Kulikowski

**Affiliations:** ^1^ Laboratório de Citogenômica, Departamento de Patologia Faculdade de Medicina FMUSP, Universidade de São Paulo São Paulo SP Brazil; ^2^ Unidade de Genética, Departamento de Pediatria, Instituto da Criança, Hospital das Clinicas HCFMUSP Faculdade de Medicina, Universidade de São Paulo São Paulo SP Brazil

**Keywords:** Brazilian patients, cri du chat, cytogenomic, genomic array

## Abstract

**Background:**

Cri du chat syndrome (CdCS) is a rare syndrome caused by a partial or complete deletion of the short arm of chromosome 5 (5p‐). The main clinical features include a high‐pitched cry, facial asymmetry, microcephaly, round face at birth, epicanthal folds, hypotonia, delayed growth and development.

**Methods:**

We studied 14 Brazilian patients with CdCS using genomic array in order to better define the 5p breakpoints and recognize copy number variations (CNVs) that contribute to clinical manifestations associated with the syndrome.

**Results:**

Array confirmed terminal deletions in 13 patients and an interstitial deletion in one patient. It was also possible to map the breakpoints and associate a genomic region of 4.7 Mb to the development of head circumference and cat‐like cry. We also found other CNVs concomitant to the 5p deletion including a 9p duplication, a 17q deletion, and a 22q deletion in three different patients.

**Conclusion:**

With advancements of molecular cytogenomic methods in the last two decades, it was possible to evidence cryptic alterations and improve the genotype–phenotype correlation. In this work, we describe a new genomic region associated with microcephaly and cat‐like cry and highlight the importance of precise delineation of 5p deletion breakpoints and detection of other CNVs in CdCS patients to improve genotype–phenotype correlation to perform a complete clinical and molecular diagnosis.

## Graphical abstract

We studied 14 Brazilian patients with Cri du chat syndrome using arrays and suggest a new genomic region associated with microcephaly and cat‐like cry. Also we highlight the importance of precise delineation of 5p deletion breakpoints for better genotype–phenotype correlation.

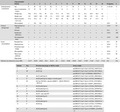

## 1 INTRODUCTION

Cri du chat syndrome (CdCS—OMIM #123450) is a genetic disorder caused by total or partial deletion of the short arm of chromosome 5, also known as 5p‐ syndrome. Lejeune and colleagues described this syndrome for the first time in 1963 when they noted newborns who presented an abnormal and high‐pitched cry, similar to a cat cry. A few years later, with the development of G‐banded karyotyping, it was possible to associate the clinical manifestations of the syndrome to the short arm of chromosome 5 (Niebuhr, 1978). The clinical symptoms include a high‐pitched cry, facial asymmetry, reduced head circumference (microcephaly), round face at birth, epicanthal folds, strabismus, laryngeal malformation, ocular hypertelorism, hypotonia, low implanted ears, prominent nasal bridge, and delayed growth and development (Mainardi et al., 2006, 2001; Niebuhr, 1978).

In the first studies, genotype–phenotype correlation was limited by the use of traditional cytogenetic techniques, such as G‐banded karyotyping and FISH (fluorescence in situ hybridization). With the advancements of molecular cytogenomic methods in the last two decades, a series of cryptic telomeric alterations revealed that many terminal deletions considered to be pure and were in fact interstitial or terminal deletions stabilized by the capture of a telomere from another chromosome (Ballif, Wakui, Gajecka, & Shaffer, 2004; Santo, Moreira, & Riegel, 2016; Yokoyama et al., 2018).

Here, we performed BeadArray technique using samples from 14 Brazilian patients with a previous CdCS diagnosis and discuss the importance of mapping all of the pathogenic copy number variants (CNVs) to better associate to specific phenotypic features.

## 2 MATERIALS AND METHODS

### 2.1 Subjects

A cohort of 14 patients with clinical diagnosis of CdCS (10 females and 4 males) were followed through clinical evaluation by geneticists at the Unit of Clinical Genetics—Instituto da Criança, Hospital das Clínicas—Universidade de São Paulo (ICr‐HCFMUSP), Brazil. The inclusion criteria were a previous clinical diagnosis of CdCS and/or cytogenetic molecular tests confirmation. The study was approved by the Institutional Review Board Ethics Committee for Analysis of Research Projects HCFMUSP/CAPPesq (CAAE: 62322416.7.0000.0068) and written consent was obtained from all the participants and their parents.

### 2.2 DNA extraction

Genomic DNA was isolated from peripheral blood lymphocytes using a commercially available DNA isolation kit (QIAamp DNA Blood Mini Kit^®^, Qiagen) in accordance with the manufacturer's instructions. The quality and quantity of the DNA samples were determined using Qubit Fluorometer (Invitrogen), and the integrity of the DNA was ascertained via agarose gel electrophoresis analysis.

### 2.3 Array analysis

A genomic array was performed for all 14 patients using the Infinium CytoSNP‐850K BeadChip, which contains approximately 850,000 selected single nucleotide polymorphisms (SNPs) with an enriched coverage for 3,262 disease‐related genes (Illumina, Inc.). The manufacturer's recommended protocol was followed and the raw data were analyzed using *BlueFuse* ^™^ *Multi* v4.4 (Illumina, Inc.). The genomic positions are given as mapped to the GRCh37/hg19 genome build.

The results were analyzed according to the American College of Medical Genetics guidelines (Kearney, Thorland, Brown, Quintero‐Rivera, & South, 2011) using independent tests and were compared with the following databanks of CNVs and classified as benign, pathogenic, or VOUS (variants of uncertain clinical significance): the Database of Genomic Variants (DGV, http://projects.tcag.ca/variation/), the Database of Chromosomal Imbalance and Phenotype in Humans Using Ensembl Resources (DECIPHER, http://decipher.sanger.ac.uk/) and the UCSC Genome Bioinformatics database (http://genome.ucsc.edu). The genomic positions are reported according to their mapping on the GRCh37/hg19 genome build.

## 3 RESULTS

We investigated 14 patients with 5p deletion syndrome who were between age 2 and 38 years. We measured height, weight, and head circumference in addition to performing the clinical examination and obtaining the previous report from the parents. The features evaluated are listed in Table 1.

**Table 1:** Main clinical features of the 14 patients and size of alterations by array according to human assembly GRCh37/hg19

	Characteristic/Patient	1	2	3	4	5	6	7	8	9	10	11	12	13	14	Frequency	%
Anthropometric examination	Sex	F	M	M	F	M	F	F	F	M	F	F	F	F	F	11 F/4 M	73
	Age at examination (years)	15	25	38	10	8	8	14	4	3	2	22	9	16	13	−	−
	Weight (kg)	30.4	67.6	NA	30	14.2	27.4	34	10.2	11	8.5	63.1	26.9	46.2	44	−	−
	Height (cm)	143	169.5	168	139	111	123.5	143	88.5	95	78	156	129	151	151	−	−
	Head circumference (cm)	47.5	55.4	51	47	43	48.5	48	44	46	43	53.5	47	47.5	52	−	−
	Microcephaly	+	NA	NA	+	+	+	+	+	+	+	NA	+	+	−	10/11	91
Clinical background	High‐pitched cry at birth	+	+	+	+	+	+	+	+	+	+	+	+	+	−	13/14	93
	Seizures	−	+	−	−	+	−	−	−	−	−	−	−	−	−	2/14	14
	Intellectual deficiency	+	+	+	+	+	+	+	+	+	+	+	+	+	+	14/14	100
	Developmental delay	+	+	+	+	+	+	+	+	+	+	+	+	+	+	14/14	100
Facial dysmorphism	Rounded face	+	−	−	−	−	−	−	+	+	+	−	−	−	−	4/14	29
	Hypertelorism	+	+	−	−	−	+	+	+	+	+	+	+	−	+	10/14	71
	Prominent nasal bridge	+	+	+	+	−	−	+	−	−	−	+	+	+	−	8/14	57
	Micrognathia	+	+	+	−	−	−	+	+	+	+	+	+	+	−	10/14	71
	Short nasal philtrum	+	+	+	+	+	−	+	−	+	+	+	+	+	+	12/14	86
	High palate	−	+	+	−	−	+	+	−	+	−	+	+	+	+	9/14	64
Deletion size detected in array (Mb)		34.37	29.94	29.83	25.6	25.32	25	22.01	19.86	18.89	18.07	17.99	17.63	17.21	17.43	NA	NA

Abbreviations: F, female; M, male; Mb, Megabase; NA, not applicable; + (positive symbol), indication of the presence of the characteristic; − (negative symbol), indication of the absence of the characteristic; % (percentage symbol), indication of the percentage of the frequency.

In this study, all the patients presented intellectual disability (ID) and minor facial dysmorphism which is typically associated with CdCS, such as hypertelorism, a prominent nasal bridge, micrognathia, a short nasal philtrum, and high palate. The values for weight and height were below the 5th percentile for most of the patients. The high‐pitched cry was present in 93.3% of the patients (Table 1).

**Figure 1:**
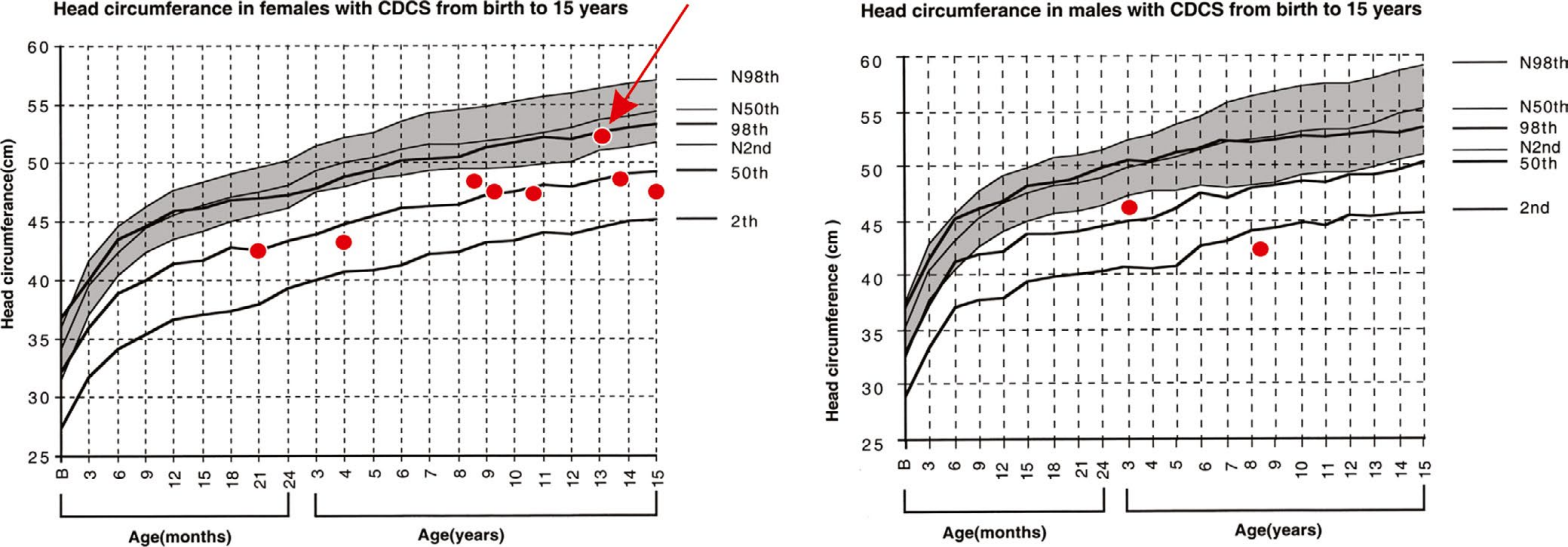
Head circumference in females and males with CdCS from birth to 15 years. Normal growth curves (N) are represented by the grey area and CdCS specific curves are indicated by the thick black lines. The results found in our 10 patients (red circles) showed that only one patient (patient 14) appears to have no microcephaly (red arrow) with measurements between 2nd < *p* < 50th for normal growth curves and 98th for CdCS’ growth curves. Adapted from Marinescu et al. (2000). CdCS, cri du chat syndrome

Head circumference was measured in 10 patients, one of whom was in the 10th percentile (*p* = 10) for head circumference (red arrow in Figure 1, between 2nd < *p*<50th for normal growth curves and in the 98th for CdCS growth curves), whereas nine had measures below the 2nd percentile for normal growth curves. Four patients were not evaluated due to the age limit of the CdCS growth charts.

### 3.1 Previous genetic results

Previous analysis using G‐banded karyotyping detected terminal deletions in 12 patients, with breakpoints between 5p13‐p15 and two patients presented normal karyotypes. For patient 9, a genetic diagnosis of CdCS was made using array. Although the patient had a prenatal karyotype without alterations, all the features resembled those of 5p‐ syndrome. Patient 5 had a previous diagnosis of 5p deletion and 9p duplication using P036 and P070 MLPA kits and FISH, as described elsewhere (Lincoln‐de‐Carvalho, Vicente, Vieira, de Mello, & Marques‐de‐Faria, 2011).

### 3.2 Array results

Array confirmed the presence of terminal deletions with distinct final breakpoints in 11 of the 12 patients with previous diagnosis using G‐banded karyotype. One patient had an interstitial deletion starting at 4,788,892 bp (base pairs) with an endpoint in 22,219,836 bp. It was also possible to confirm the 5p deletion and 9p duplication in patient 5 and estimate the size of both CNVs: the chromosome 5 breakpoints were at 25,328 bp and 25,351,609 bp, and the chromosome 9 breakpoints were at 46,587 bp (position of the first probe from the BeadChip in 9p) and 20,642,438 bp.

Evaluating the breakpoints, we have observed that the 13 patients had initial breakpoint at 25,328 bp (position of the first probe from the used BeadChip in 5p) and all the patients had distinct end breakpoints between 17,235,998 bp and 34,376,825 bp.

We also found other CNVs concomitant to the 5p deletion including a 9p duplication, a 17q deletion, and a 22q deletion in three different patients.

It was possible to redefine the size of all deletions, as shown in Table 2.

**Table 2:** Results obtained by a previous test (karyotype or MLPA) compared to results obtained by array, according to GRCh37/hg19, including CNVs’ breakpoints found in array

Patient	Sex	Previous karyotype or MLPA result	Array results
1	F	46,XX,5p‐	arr[GRCh37] 5p15.33p13.2(25328_34402152)x1
2	M	46,XY,5p‐	arr[GRCh37] 5p15.33p13.3(25328_29971508)x1
			arr[GRCh37] 17q21.3(43996960_44002555)x1
3	M	46,XY,del(5)(p13)	arr[GRCh37] 5p15.33p13.3(25328_29863566)x1
4	F	46,XX,del(5)(p14),14pstk+,15cenh+pstk+	arr[GRCh37] 5p15.33p14.1(25328_25658882)x1
5	M	Del 5p (*PDCD6*) e dup 9p (*DMRT1*) – MLPA P036‐E1/P070‐A2	arr[GRCh37]5p15.33p14.1(25328_25351609)x1
			arr[GRCh37] 9p24.3p21.3(46587_20642438)x3
6	F	46,XX,del(5)(p14)	arr[GRCh37] 5p15.33p14.1(25328_25027618)x1
7	F	46,XX,del(5)(p14.2)	arr[GRCh37] 5p15.33p14.3(25328_22039679)x1
8	F	46,XX,del(5)(p14.2)	arr[GRCh37] 5p15.33p14.3(25328_19892934)x1
9	M	46,XY—pre‐natal KT	arr[GRCh37] 5p15.33p14.3(25328_18921988)x1
10	F	46,XX,del(5)(p15)	arr[GRCh37] 5p15.33p15.1(25328_18099766)x1
11	F	46,XX,5p‐	arr[GRCh37] 5p15.33p15.1(25328_18022107)x1
12	F	46,XX,del(5)(p14)	arr[GRCh37] 5p15.33p15.1(25328_17656351)x1
13	F	46,XX,5p‐	arr[GRCh37] 5p15.33p15.1(25328_17235998)x1
			arr[GRCh37] 22q12.3(36753803_36782997)x1
14	F	46,XX,del(5)(p14)	arr[GRCh37] 5p15.32p14.3(4788892_22219836)x1

Abbreviation: CNVs, copy number variants.

**Figure 2:**
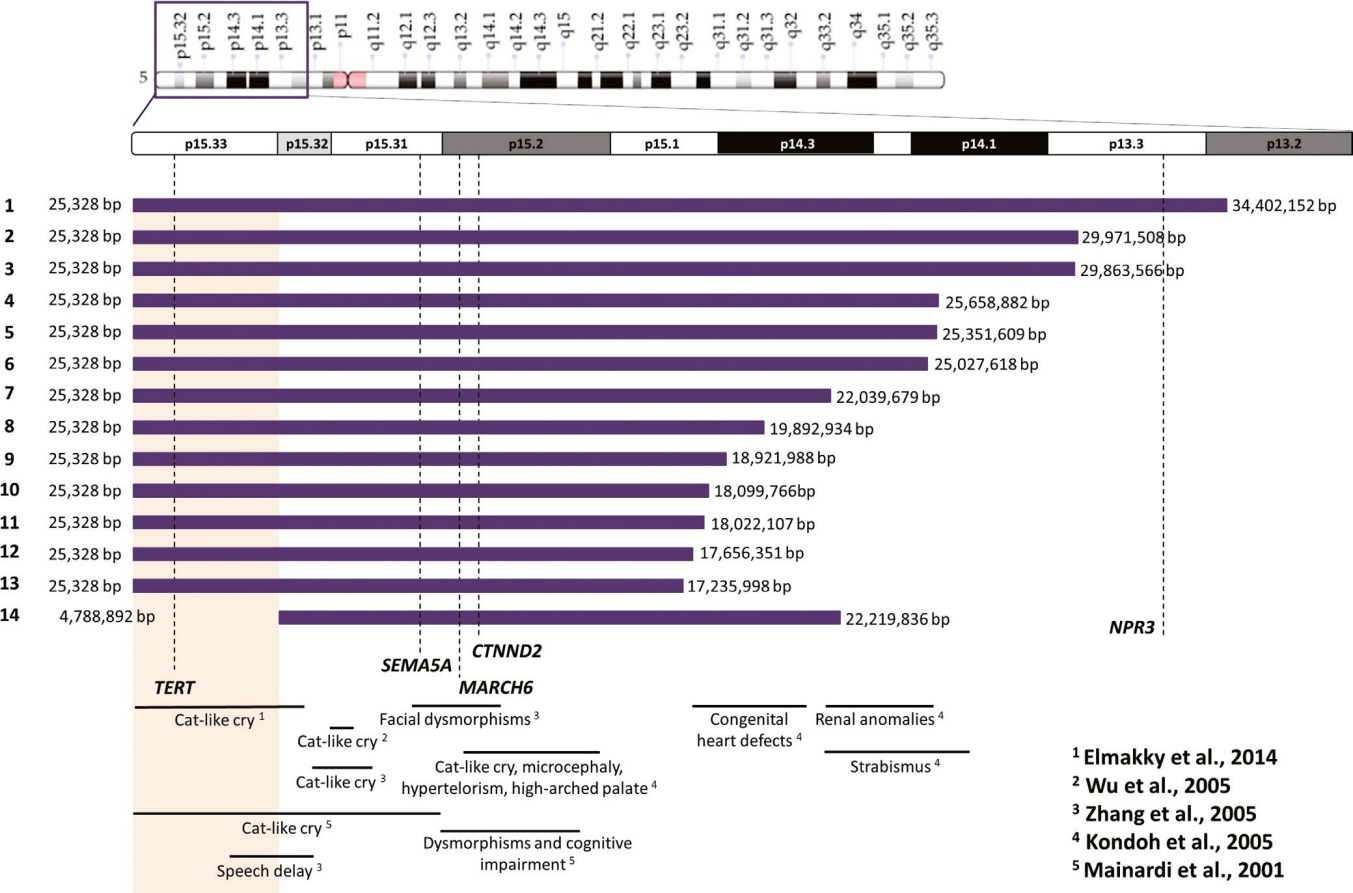
Representation of the deletions extent, indicated by the horizontal purple bars, and breakpoints in basepairs detected through array. *TERT*, *SEMA5A*, *MARCH6*, *CTNND2*, and *NPR3* are marked with dotted lines. At the bottom are presented the genotype–phenotype relationships from data in previous publications. The genomic region we propose to be associated with head circumference and cat‐like cry is indicated in the orange box. p, short arm; q, long arm; bp, base pairs

The range of the genomic breakpoints varied widely: the smallest deletion encompassed 17.21 Mb and the largest spanned 34.37 Mb (Figure 2). None of the patients had the same initial and final breakpoints.

### 3.3 Haploinsufficient genes

Nguyen et al. (2015) classified five genes as haploinsufficient and phenotype‐related in CdCS. These genes are *TERT*, *SEMA5A*, *MARCH6*, *CTNND2*, and *NPR3*. Figure 2 indicates that three of these genes (*SEMA5A*, *MARCH6*, and *CTNND2*) were deleted in all of the studied patients, *TERT* was deleted in all patients except patient 14, and *NPR3* was deleted only in patient 1 (Figure 2).

## 4 DISCUSSION

Although genomic array is the tool of choice for investigation in patients with congenital malformations and intellectual disabilities, the high cost of this methodology for the Brazilian public health‐care system (SUS—Sistema Único de Saúde) greatly hinders the implementation, especially due to economic constraints. Therefore, G‐banded karyotype is the only test available for most of Brazilian patients with clinical manifestations of CdCS, and several cases are still inconclusive (Zanardo et al., 2017).

Since molecular diagnosis is not available for most of the population, clinicians evaluate minor dysmorphologies in order to provide a more accurate diagnosis. The values for weight and height for most of the patients in this study were below the 5th percentile, in accordance with Marinescu et al. (2000). A high‐pitched cry was present in almost all the patients (93.3%), being the most easily identifiable feature (Niebuhr, 1978).

Seizures were identified in two patients (13.3%), patient 5 and patient 2. There is a lack of information about the relation of seizures and CdCS; however, this is a rare feature in CdCS patients (Nakagami, Terada, Ikeda, Hiyoshi, & Inoue, 2015). Niebuhr (1978) described seizures in 2.7% of a 331‐patient cohort, a considerably lower frequency than the one found in this study (14%). The most likely explanation for this discrepancy is that brain disorders may be associated with other variables which do not involve 5p and have not yet been investigated in previous studies.

Indeed, in patient 5, seizures may be linked to trisomy 9p. This trisomy is associated with cerebral, neurological, and epileptic abnormalities (Nakayama et al., 2012; Stern, 1996). Array revealed that patient 2 presented a small deletion located at 17q21.3. This region is in the *MAPT* associated with frontotemporal dementia (OMIM #600274), a feature the patient presented during neurological testing. This deletion was classified as a variant of uncertain significance (VUS). Another reason for the low occurrence of seizures in CdCS patients could be the size of the cohort of this study. This possibility highlights the need for further investigation of other variables that may affect this finding.

Microcephaly (considered as head circumference below the 3rd percentile) and cat‐like cry were present in all patients except patient 14. Patient 14 was also the only patient in this study with an interstitial deletion. Combining these results, we could suggest a region associated with the presence of microcephaly and the cat‐like cry. This genomic region would be narrowed to the 4.7 Mb between 25,328 bp and 4,788,892 bp, which were the breakpoints detected in patient 14 (Figure 2). In accordance with our results, Elmakky et al. (2014) also suggested that a terminal region of 5.5 Mb found between 5p15.32–33 would regulate the growth of the head throughout life and is also related to other dysmorphic features.

However, there is conflicting information from previous studies. Kondoh et al. (2005) inferred that the region associated with cephalic perimeter was between 10 and 15 Mb and was also related to other features without genomic position distinction. This discrepancy can be explained by the fact that the authors had an *n* = 6 and used only karyotype and FISH with specific chromosome 5 probes, without analyzing other CNVs that could interfere with this phenotype.

Evaluating the high‐pitched cry specifically, multiple regions have been reported for this feature (Figure 2). This feature was related to the 5p15.2 region with the involvement of *MARCH6* (Gersh et al., 1995; Overhauser et al., 1994). However, Wu, Niebuhr, Yang, and Hansen (2005) attributed this feature to *FLJ25076*, which is involved in *MARCH6* degradation signaling pathway, and is located between 6,365,349 and 7,003,686 bp. *FLJ25076* is locally expressed in thoracic and scalp tissues, which could explain the cat‐like cry. Zhang et al. (2016) were able to delineate a critical region of approximately 700 kb at 5p15.32, which includes only two coding genes, *ADAMTS16* and *ICE1*.

Multiple critical regions have been reported for microcephaly and the high‐pitched cry in patients with 5p‐ syndrome, but it was always present with other features, and the use of different molecular techniques makes it difficult to delineate a genomic region corresponding to these particular phenotypes.

In addition to the cat‐like cry, facial dysmorphism is also indicative of the syndrome. Despite the existence of descriptions in the literature regarding changes in the phenotype of 5p‐ patients between childhood and adulthood (in older CdCS patients, the phenotype resembles that of Angelman Syndrome), there is no precise age reported for this change (Santo et al., 2016; Van Buggenhout et al., 2000).

Therefore, we suggest that the transition from a round face to an elongated face occurs between 4 and 8 years. Patients aged 2–4 years had a round face and older patients (over 8 years) had a more elongated face, with the exception of patient 1. In patient 1, the face traits were inherited from the patient's mother, who also has the same features. Revisiting a Brazilian website focused on pictures and reports of parents of CdCS patients (https://www.portalcriduchat.com.br), we found pictures of patient 13 at a younger age demonstrating the rounded face and supporting our hypothesis.

Emerging tools, such as Face2Gene application, can help to identify a possible match with this syndrome for patients with ID. We tested this application and detected that in 80% of the cases CdCS was one of the three main suggested syndromes.

In the present study, all of the patients had ID and were able to speak few words, communicate with gestures, and use aggressive behavior as a way to demonstrate dissatisfaction. Using a numerical scale for ID that ranged from 0 (unaffected) to 7 (profoundly affected), adapted from Zhang et al. (2005), we observed that 12 patients were classified as severe or very severe and two were classified as moderate.

Those two patients (patients 4 and 13) were able to communicate and express themselves through words and sentences, although with some difficulty. Evaluating the size of the deletion on chromosome 5, we observed there was great divergence between the deletion sizes (17.21 Mb and 25.6 Mb for patients 13 and 4, respectively). These data suggest the significant clinical variability in intellectual development in CdCS syndrome and no relation between deletion size and ID (Marinescu, Johnson, Dykens, Hodapp, & Overhauser, 1999; Marinescu, Johnson, Grady, Chen, & Overhauser, 1999).

Array results also revealed other CNVs not related to 5p. We found three CNVs concomitant to the 5p deletion: a pathogenic 9p duplication, a 17q deletion classified as a VUS, which was associated with frontotemporal dementia, and a 22q deletion as probably pathogenic. The deletion in 22q in patient 13 was classified as being probably pathogenic since the patient had hearing impairment, and this region is associated with the *MYH9*, which is responsible for cochleosaccular degeneration (OMIM #603622).

The phenotype of CdCS patients results from the deletion of several genes located in 5p and in some cases the addition of other CNVs. Although chromosome 5 is one of the chromosomes with the lowest gene density, it covers genes responsible for aging, facial dysmorphisms, and neuronal development. The haploinsufficiency of the genes in the region is responsible for the known phenotype and for the great variability in the development of these patients (Nguyen et al., 2015).

*SEMA5A* and *CTNND2*, deleted in all patients, are related to brain development and migration of neurons. The hemizygosity of these genes can complicate this process and lead to ID, reported in all patients in this study, and autistic spectrum behaviors (Duan et al., 2014; Medina, Marinescu, Overhauser, & Kosik, 2000; Zhang et al., 2016).

All of the patients had deletion of *TERT* gene, except patient 14. Heterozygous deletion of *TERT* is related to failures in telomere length maintenance and phenotypic changes in adults (Zhang et al., 2003). We could not conclude whether the presence of *TERT* in patient 14 was phenotypically relevant since she was not seen at a younger age and did not present gray hair at the clinical evaluation.

Another gene classified as dosage‐sensitive is *NPR3*, deleted in one patient. A study in rats showed that this gene seems to be related to hypertension and blood pressure increase regardless of salt intake, but the effect in humans remains uncertain (Kuang, Zhou, Li, Tang, & Chen, 2017). Deletion of this gene did not show any physiological impact.

In this work, we describe a new genomic region associated with microcephaly and cat‐like cry and highlight the importance of precise delineation of 5p deletion breakpoints and detection of other CNVs in CdCS patients to improve genotype–phenotype correlation to perform a complete clinical and molecular diagnosis.

## CONFLICT OF INTEREST

There are no conflict of interest to disclose.
